# Next-generation sequencing yields the complete chloroplast genome of *Abies yuanbaoshanensis*, an endangered species from South China

**DOI:** 10.1080/23802359.2020.1840938

**Published:** 2020-12-24

**Authors:** Yuan-Yuan Zhang, Rui-Chen Xiang, Na-Lin Dong, Yan-Yan Liu, Yi-Zhen Shao

**Affiliations:** a College of Life Sciences, Henan Agriculture University, Zhengzhou, PR China; b College of Forestry, Henan Agriculture University, Zhengzhou, PR China; c College of Plant Protection, Henan Agriculture University, Zhengzhou, PR China

**Keywords:** *Abies yuanbaoshanensis*, chloroplast genome, endangered, phylogenetic

## Abstract

*Abies yuanbaoshanensis* is critically endangered and restricted in the Yuanbao Mountain of China, with no more than 900 surviving individuals. Here, we reported the complete chloroplast (cp) genome of *A. yuanbaoshanensis*. The complete chloroplast genome is 121,795 bp in size. In total, 114 genes were identified, including 68 peptide-encoding genes, 35 tRNA genes, 4 rRNA genes, 6 open reading frames, and 1 pseudogene. Thirteen genes contain introns. In phylogenetic analysis, both the ML and BI analyses supported the monophyly of the genus *Abies*. Our study will provide potential genetic resources for further conservation and evolutionary studies of this highly endangered species.

The fir genus *Abies* Miller species are ecologically important because they are a major component of the cold temperate forests and provide a basic home for a great diversity of animals and plants (Liu 1971; Farjon 1990, 2001). However, many firs have been listed as endangered species (Xiang et al. 2015). *Abies yuanbaoshanensis* Y. J. Lu & L. K. Fu is listed as highly endangered species in the Red List (IUCN 2018). At present, *A*. *yuanbaoshanensis* is restricted in the Yuanbao Mountain (Guangxi province, China), with less than 900 surviving individuals. Here, we assembled and characterized the complete plastome of *A*. *yuanbaoshanensis*. It will provide potential genetic resources for further conservation and evolutionary studies of this highly endangered fir species.

The plant material of *A. yuanbaoshanensis* was collected from a single individual that lives in the natural forest habitat of Mt. Yuanbao (25.39°N, 109.17°E). Voucher specimen and DNA sample (Xiang Q.-P., No. YB) were deposited in the herbarium of Institute of Botany, CAS (PE). Total genome DNA was extracted with the Ezup plant genomic DNA prep kit (Sangon Biotech, Shanghai, China). Total DNA was used to generate libraries with an average insert size of 350 bp, which were sequenced using the Illumina HiSeq X platform. In total, ca. 10.1 million high-quality clean reads (150 bp PE read length) were generated with adaptors trimmed. The CLC *de novo* assembler (CLC Bio, Aarhus, Denmark), BLAST, GeSeq (Tillich et al. 2017), and tRNAscan-SE version 1.3.1 (Schattner et al. 2005) were used to align, assemble, and annotate the plastome. Genome annotation was performed by comparing the sequences with the cp genomes of *Abies koreana* (KP742350) and *A. neprolepis* (KT834974).

The full length of *A. yuanbaoshanensis* chloroplast genome (GenBank Accession No. MH706718) was 1,21,795 bp. The chloroplast genome showed a typical quadripartite structure that consisted of a pair of IR regions (264 bp) separated by the LSC (67,105 bp) and SSC (54,162 bp) regions, which was similar to the majority of cp genomes in Pinaceae. The GC content was 38.30%. A total of 114 genes were contained in the cp genome (68 peptide-encoding genes, 35 tRNA genes, 4 rRNA genes, 6 open reading frames, and 1 pseudogene). A total of 53 protein-coding, 16 tRNA genes, 3 open reading frames, and 1 pseudogene are located in the LSC region, while 15 protein-coding, 17 tRNA genes, 4 rRNA, and 3 open reading frames are located in the SSC region, respectively. Only one tRNA gene (*trnI-CAU*) is duplicated and located on the IR regions. All *ndh* genes have been lost in the genome of *A*. *yuanbaoshanensis* like other cp genomes of family Pinaceae. Among the protein-coding genes, two genes (rps12 and ycf3) contained two introns, and other 11 genes (*trnK-UUU*, *trnV-UAC*, *rpoC1*, *atpF*, *trnG-GCC*, *petB*, *petD*, *rpl16*, *rpl2*, *trnL-UAA*, and *trnA-UGC*) had one intron each. In previous studies, short inverted repeat sequences which consist of trnS-psaM-ycf12-trnG and trnG-ycf12-psaM-trnS (1183 bp) are located in 52-kb inversion points of the cp genome of *A*. *yuanbaoshanensis*. Length and sequence of inverted repeats from *A*. *yuanbaoshanensis* are identical with those of *A. koreana* (Yi et al. 2015).

Five chloroplast genomes were selected to infer the phylogenetic relationships among the main representative species of Pinaceae with *Ginkgo biloba* (Ginkgoaceae) as the outgroup. These sequences were fully aligned with MAFFT version 7.3 (Suita, Osaka, Japan) (Katoh and Standley 2013). For conducting maximum likelihood (ML) analyses, the ML inference was performed using GTRþIþC model with RAxML version 8.2.1 (Karlsruhe, Germany) (Stamatakis 2014) on the CIPRES cluster service (Miller et al. 2010). We also used MrBayes version 3.2.2 (Stockholm, Swedish) (Ronquist and Huelsenbeck 2003; Ronquist et al. 2012) for the Bayesian inference analyses. MrBayes was run for 1,000,000 generations, sampling and printing every 100 generations. Our phylogenetic analyses yielded largely congruent topologies by the ML and BI analyses. Based on these six cp genome sequences, the three *Abies* species (*A. yuanbaoshanensis*, *A. koreana*, and *A. neprolepis*) are supported as one monophyletic lineage with extremely high probabilities (BS_ML _= 100, BI_PP _= 1.0) (Figure 1).

**Figure 1.**
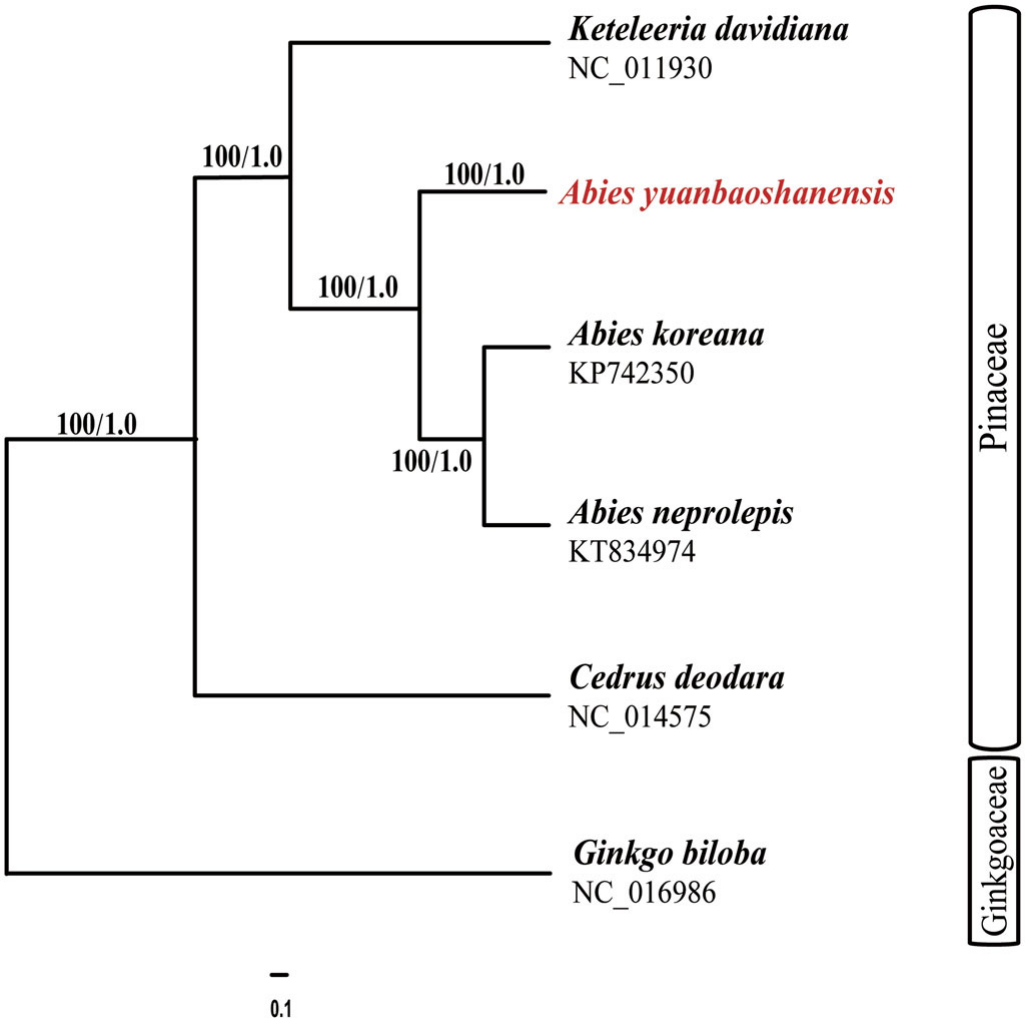
Phylogram of *Abies yuanbaoshanensis* obtained from the maximum likelihood analysis of the whole chloroplast genome sequences. Numbers on branches are support values [maximum likelihood bootstrap values (BS_ML_)/Bayesian inference posterior probability values (PP_BI_)].

This study provides new insight into the cp genome evolution and phylogenetic relationships of high endangered species. Moreover, it would be fundamental to formulate potential conservation and management strategies for this endangered species of south China.
